# Prevalence and patterns of antibiotic prescriptions in Conakry hospitals, Guinea: a multicentre cross-sectional survey

**DOI:** 10.1093/jacamr/dlaf223

**Published:** 2025-11-20

**Authors:** Thibaut Armel Chérif Gnimadi, Kadio Jean-Jacques Olivier Kadio, Aïssata Camara, Castro Gbêmêmali Hounmenou, Saidouba Cherif Camara, Salifou Talassone Bangoura, Elsie Hermine Ogoumma, Alpha Kabiné Keita, Djiba Kaba, Tiguidanke Camara, Yamoussa Youla, Amadou Daye Diallo, Mamadou Saliou Sow, Mano Joseph Mathew, Alpha Kabinet Keita, Abdoulaye Touré

**Affiliations:** Centre de Recherche et de Formation en Infectiologie de Guinée (CERFIG), Université Gamal Abdel Nasser de Conakry, Conakry, Guinea; Laboratoire de Génomique, Bioinformatique et Chimie Moléculaire, EA7528, Conservatoire National des Arts et Métiers, HESAM Université, 2 Rue Conté, Paris 75003, France; Centre de Recherche et de Formation en Infectiologie de Guinée (CERFIG), Université Gamal Abdel Nasser de Conakry, Conakry, Guinea; Faculté des Sciences et Techniques de la Santé, Université Gamal Abdel Nasser de Conakry, Conakry, Guinea; Faculté des Sciences et Techniques de la Santé, Université Gamal Abdel Nasser de Conakry, Conakry, Guinea; Parasitology Unit, Institut Pasteur of Guinea, Conakry, Guinea; Centre de Recherche et de Formation en Infectiologie de Guinée (CERFIG), Université Gamal Abdel Nasser de Conakry, Conakry, Guinea; Université de Labé, Département Informatique, Labé, Guinea; Centre de Recherche et de Formation en Infectiologie de Guinée (CERFIG), Université Gamal Abdel Nasser de Conakry, Conakry, Guinea; Centre de Recherche et de Formation en Infectiologie de Guinée (CERFIG), Université Gamal Abdel Nasser de Conakry, Conakry, Guinea; Faculté des Sciences et Techniques de la Santé, Université Gamal Abdel Nasser de Conakry, Conakry, Guinea; Centre de Recherche et de Formation en Infectiologie de Guinée (CERFIG), Université Gamal Abdel Nasser de Conakry, Conakry, Guinea; Centre de Recherche et de Formation en Infectiologie de Guinée (CERFIG), Université Gamal Abdel Nasser de Conakry, Conakry, Guinea; Faculté des Sciences et Techniques de la Santé, Université Gamal Abdel Nasser de Conakry, Conakry, Guinea; Centre de Recherche et de Formation en Infectiologie de Guinée (CERFIG), Université Gamal Abdel Nasser de Conakry, Conakry, Guinea; Faculté des Sciences et Techniques de la Santé, Université Gamal Abdel Nasser de Conakry, Conakry, Guinea; Centre de Recherche et de Formation en Infectiologie de Guinée (CERFIG), Université Gamal Abdel Nasser de Conakry, Conakry, Guinea; Centre de Recherche et de Formation en Infectiologie de Guinée (CERFIG), Université Gamal Abdel Nasser de Conakry, Conakry, Guinea; Faculté des Sciences et Techniques de la Santé, Université Gamal Abdel Nasser de Conakry, Conakry, Guinea; Service des Maladies Infectieuses et Tropicales, Hôpital National Donka, Conakry, Guinea; Centre de Recherche et de Formation en Infectiologie de Guinée (CERFIG), Université Gamal Abdel Nasser de Conakry, Conakry, Guinea; Service des Maladies Infectieuses et Tropicales, Hôpital National Donka, Conakry, Guinea; Laboratoire de Génomique, Bioinformatique et Chimie Moléculaire, EA7528, Conservatoire National des Arts et Métiers, HESAM Université, 2 Rue Conté, Paris 75003, France; EFREI Research Lab, Panthéon Assas University, 30-32 Avenue de la République, Villejuif 94800, France; Centre de Recherche et de Formation en Infectiologie de Guinée (CERFIG), Université Gamal Abdel Nasser de Conakry, Conakry, Guinea; Faculté des Sciences et Techniques de la Santé, Université Gamal Abdel Nasser de Conakry, Conakry, Guinea; Centre de Recherche et de Formation en Infectiologie de Guinée (CERFIG), Université Gamal Abdel Nasser de Conakry, Conakry, Guinea; Faculté des Sciences et Techniques de la Santé, Université Gamal Abdel Nasser de Conakry, Conakry, Guinea

## Abstract

**Background:**

Inappropriate use of antibiotics is a major driver of antimicrobial resistance (AMR), particularly in low- and middle-income countries. Understanding antibiotic use and patterns in hospital settings is essential for promoting rational use and optimizing antimicrobial stewardship (AMS). This study aims to assess the extent of antibiotic prescribing in secondary and tertiary hospitals in Conakry, Guinea, and to evaluate the appropriateness of these prescriptions on the basis of WHO recommendations via the Access, Watch, Reserve (AWaRe) classification of antibiotics.

**Methods:**

A multicentre cross-sectional survey was conducted from June to October 2024 to assess patient antibiotic use levels across six hospital wards in Conakry, capital of Guinea. The prevalence of antibiotic prescriptions, with 95% confidence intervals (CI), was compared across patient, prescriber and ward variables. Antibiotic use was categorized by Anatomic Therapeutic Chemical and AWaRe classifications. Associations between categorical variables were assessed using the Chi-square or Fisher's exact test. Univariate and multivariate logistic regression were used to analyse factors associated with antibiotic prescription.

**Results:**

Of 1482 patients surveyed, the overall prevalence of antibiotic prescriptions was 35.0% (95% CI: 32.6–37.5), with significant differences between inpatients (83.4%, 95% CI: 78.1–87.6) and outpatients (25.1%, 95% CI: 22.7–27.6). The total number of antibiotics prescribed was 669, and the most commonly prescribed antibiotics were beta-lactams/beta-lactamase inhibitors (24.2%), followed by third-generation cephalosporins (21.7%), imidazoles (18.5%), and penicillins (13.6%). Almost all antibiotic courses (99.4%) were started empirically, without microbiological testing to guide choice. Regarding the AWaRe classification of all prescribed antibiotics, Access antibiotics accounted for 64.3% (430/669), and 33.3% (223/669) were from the Watch group.

**Conclusions:**

The results of this study, conducted in six hospital departments, provide an overview of antibiotic prescriptions in Conakry hospitals, with a high prevalence of antibiotic prescription, particularly among inpatients and almost all courses were initiated empirically without microbiological guidance. These findings underscore the urgent need for AMS programs and interventions.

## Introduction

Antimicrobial resistance (AMR) is a serious public health problem worldwide, and it is considered one of the top 10 threats to global public health, and is projected to cause nearly 39 million deaths between 2025 and 2050.^1^ According to a report published in 2019, almost 1.27 million deaths were associated with antibiotic-resistant bacterial infections, much higher than the death rate attributed to HIV or malaria.^2^ The highest burden of deaths attributable to AMR organisms is observed in sub-Saharan Africa.^2,3^

Beyond regional disparities, the global public health impact of excessive, inappropriate and unnecessary antimicrobial use has been demonstrated by the risks of the development and emergence of pathogen resistance to antimicrobials, increased hospitalization rates, increased treatment costs, adverse events, and wasted resources.^4,5^ Despite this existing evidence, the use of antibiotics in resource-limited countries is weakly regulated and poorly tracked.^6^ Antibiotic consumption increased by 65% between 2000 and 2015, with the most significant increases observed in low- and middle-income countries, where antibiotics are prescribed to 25%–50% of patients and may represent up to 30% of total medication expenditures.^7,8^ Studies have also shown that over 50% of outpatient prescriptions are dispensed inappropriately.^9^ Between 20% and 50% of all antibiotics prescribed in acute care hospitals are unnecessary or inappropriate.^10,11^

In Guinea, a lower-middle-income country in West Africa with an estimated population of 14 million, evidence shows that the prevalence of antibiotic prescriptions in healthcare facilities varies by context and level of care. Conakry, the capital city, concentrates most tertiary-level hospitals, while rural areas remain largely underserved. The country adopted a National Action Plan on antimicrobial resistance in line with the WHO Global Action Plan,^12^ although implementation and antimicrobial stewardship activities remain very limited. The healthcare system is predominantly financed through out-of-pocket payments, with minimal government-funded insurance coverage.^13^ A study carried out in rural areas revealed that 62.9% of febrile patients were prescribed at least one antibiotic,^14^ while another study in an urban setting, in one department of a national hospital in Conakry, reported an antibiotic prescription frequency of 21%.^15^

Many prescriptions of antibiotics are not aligned with evidence-based guidelines.^16^ To address this issue and mitigate the global threat of AMR, WHO has developed initiatives aimed at supporting countries and hospitals in monitoring antibiotic use, promoting appropriate prescribing practices and evaluating the effectiveness of antimicrobial stewardship programs. Among these initiatives are the establishment of the Global Antimicrobial Resistance and Use Surveillance System (GLASS) in 2015 and the introduction of the Access, Watch, and Reserve (AWaRe) antibiotic classification framework in 2017.^17–19^ Under the AWaRe framework, Access antibiotics includes narrow-spectrum antibiotics recommended as first- and second-line empiric treatments for the most common clinical syndromes.^20^ Watch antibiotics generally include broad-spectrum antibiotics, which are critically important for human medicine but have a greater potential to cause AMR and thus should be used for critically ill patients in hospital settings. The Reserve antibiotics include antibiotics used as a last resort and are reserved for multidrug-resistant pathogens.^20^ WHO recommends that at least 70% of national antibiotic consumption should come from Access group antibiotics, with minimal use of Reserve antibiotics.^21^ By stratifying antibiotics into Access, Watch, and Reserve groups, the AWaRe tool guides prescribers and policymakers towards rational prescribing.^22^ This framework promotes rational use, supports antimicrobial stewardship (AMS), and facilitates monitoring of antibiotic consumption at national and global levels.^23^ Despite efforts to promote and operationalize these tools, implementation and integration remain a challenge in many resource-limited countries.

Consequently, ongoing monitoring on the basis of descriptive studies of their frequency of use is essential to understand how they are prescribed and used in line with recommendations. In these times of emerging infectious diseases, such an approach seems crucial to prevent the emergence of bacterial resistance, guarantee the effectiveness of treatments and protect the health of the population. We aimed to assess the extent of antibiotic prescribing in secondary and tertiary hospitals in Conakry, Guinea, and to evaluate the appropriateness of these prescriptions on the basis of WHO recommendations via the AWaRe classification of antibiotics.

## Materials and methods

### Study design, setting, and approach

This study was conducted in two health facilities in Conakry, Guinea, where public healthcare comprises health posts and health centres (primary level), prefectural and regional hospitals (secondary level) and national hospitals (tertiary level). The study focused on secondary and tertiary levels.

At the secondary level, data were collected from the Municipality Medical Centre Matam (CMC/Matam), specifically within the general medicine and gynaecology wards. At the tertiary level, the study was conducted in two national hospitals, the National Hospital of Donka and the Sino-Guinean Friendship Hospital, within the dermatology, traumatology, infectious disease and emergency wards.

This was a cross-sectional study including both inpatients and outpatients who received their first antibiotic prescription after visiting the wards. Outpatients were eligible if they attended the department between 8:00 a.m. and 2:00 p.m., whereas inpatients were eligible if they had been admitted the previous day or before 8:00 a.m. and had received their first antibiotic prescription during that period. Patients who had been hospitalized for several days and were already receiving antibiotic treatment, as well as those with known histories of HIV infection or tuberculosis, were excluded.

### Sampling size and data collection

The sample size was calculated based on patients as the unit of analysis. For each department, the Schwartz formula was applied using the reported frequency of antibiotic prescription from a previous study conducted in an internal medicine ward in Guinea,^15^ with a 10% margin of error. This resulted in a required sample size of 280 patients per ward, applied to both levels of facility included in the study.

Data were collected from June to October 2024 via individual survey forms supplemented by direct patient interviews and information extracted from medical records and prescriptions. The data collection tool was developed based on a modified WHO Methodology for Point Prevalence Surveys (WHO-PPS) on Antibiotic Use in Hospitals, version 1.1.^24^

Before full implementation, two final-year pharmacy students were trained on the questionnaire and data collection procedures. Pharmacy students were selected as data collectors due to their background knowledge of antibiotics. A pilot test was conducted to refine the tool and ensure its appropriateness and reliability.

### Data analysis

Descriptive statistics were used to summarize the characteristics of patients, healthcare providers, healthcare facilities and prescribed antibiotics. The prevalence of antibiotic prescriptions, distribution of antibiotic characteristics by patients and prescribers, and information by hospital wards and AWaRe classification were determined at the patient level (i.e. the number of participants with at least one antibiotic prescribed). The proportion of prescribed antibiotics, AWaRe antibiotics, classification by classes and distribution across hospital wards and patients’ status at the antibiotic level (total number of individual prescribed antibiotics).

Comparisons were made by using the Chi-square test. For contingency tables including small, expected cell counts, particularly in the analysis of antibiotics distribution by patient status and across hospital wards, Fisher's exact test with Monte Carlo simulation (10 000 replicates) was used to ensure the robustness of the statistical inference. Factors associated with antibiotic prescription were analysed with univariate and multivariate logistic regression.

A significance level of 0.05 was considered, and results were interpreted at a 95% Confidence intervals (CI). Finally, we use the WHO ATC and AWaRe classification^17^ to categorize and evaluate the practice of antibiotic prescription in this study. All analyses were performed using R statistical software (version 4.4.1).^25^

### Ethical consideration and consent to participate

The study was approved by the National Ethics Committee for Health Research of Guinea (Comité National d’Éthique pour la Recherche en Santé—CNERS) under registration number **127/CNERS/24**. Before data collection, official authorization was obtained from the administrative leadership of each participating hospital.

All participants were provided with information and a consent form that explained the purpose of the study. Patients were included only after providing their informed consent, in accordance with the principles of ethical research.

## Results

### Demographic characteristics of patients, prescribers and hospital structures

A total of 1482 patients received medication prescriptions. The patient's age ranged from 1 month to 97 years, with a mean age of 35.5 ± 18.7 years. Females accounted for 57.6% of all participants. These patients were recruited from six different departments as follows: 19.6% (291/1482) from dermatology, 19.9% (295/1482) from emergency medicine, 8.4% (124/1482) from general medicine, 18.7% (277/1482) from gynaecology, 19.8% (294/1482) from traumatology and 13.6% (201/1482) from infectious diseases. The majority of patients were seen in outpatient consultations (82.9%, *n* = 1229/1482), whereas 17% (*n* = 253/1482) were hospitalized.

Regarding the prescriber profiles, general practitioners represented 60% (*n* = 888/1482), followed by specialists (24.5%, *n* = 363/1482) and medical interns (24.5%, *n* = 230/1482). Furthermore, 71.3% (*n* = 1056/1482) of prescribers had more than 5 years of professional experience (Table 1).

**Table 1.** Characteristics of patients, prescribers, and hospitals with antibiotic prescribing in Conakry, Guinea (June–October 2024)

Characteristic	*n* (%)	Antibiotics prescribing		*P* value
		No *n* (%)	Yes *n* (%)	
Overall	1482	963 (65.0)	519 (35.0)	
Sex				0.030
Male	629 (42.4)	389 (61.8)	240 (38.2)	
Female	853 (57.6)	574 (67.3)	279 (32.7)	
Age group				0.4
1–23 months	13 (0.89)	8 (61.5)	5 (38.6)	
2–18 years	177 (12.16)	106 (59.9)	71 (40.1)	
18–49 years	949 (65.2)	627 (66.0)	322 (34)	
>50 years	317 (21.7)	207 (65.3)	110 (34.7)	
Occupation				0.2
Civil servant	300 (20.2)	197 (65.7)	103 (34.3)	
Marketer	182 (12,3)	124 (68.1)	58 (31.9)	
Self-employed professional	597 (40.3)	397 (66.5)	200 (33.5)	
Unemployed	403 (28.2)	245 (60.8)	158 (39.2)	
Study level				0.6
Educated	896 (60.5)	577 (64.4)	319 (35.6)	
No educated	586 (39.5)	386 (65.9)	200 (34.1)	
Marital status				0.047
Married	913 (61.6)	611 (67.0)	302 (33.0)	
Single	569 (38.4)	352 (61.9)	217 (38.1)	
Patient status				<0.001
Outpatient	1 229 (82.9)	921 (74.9)	308 (25.1)	
Inpatient	253 (17.0)	42 (16.6)	211 (83.4)	
Prescriber				<0.001
Specialist	363 (24.5)	198 (54.55)	165 (45.45)	
General practitioner	888 (60.0)	605 (68.1)	283 (31.9)	
Medical interns	230 (15.5)	160 (69.6)	70 (30.4)	
Prescribers’ experience				0.2
<5 years	426 (28.7)	288 (67.6)	138 (32.4)	
>5 years	1056 (71.3)	675 (64)	381 (36.0)	
Prescription wards				<0.001
Dermatology	291 (19.6)	211 (72.5)	80 (27.5)	
Emergency	295 (19.9)	174 (59)	121 (41.0)	
General medicine	124 (8.4)	100 (80.65)	24 (19.35)	
Gynaecology	277 (18.7)	178 (64.3)	99 (35.7)	
Traumatology	294 (19.8)	146 (49.7)	148 (50.3)	
Infectious diseases	201 (13.6%)	154 (76.6)	47 (23.4)	
Hospital level				0.033
Secondary hospital	401 (27.1)	278 (69.3)	123 (30.7)	
Tertiary hospital	1081 (72.9)	685 (63.4)	396 (36.6)	

### Prevalence of antibiotic use

Among the 1482 patients included, 519 (35.0%; 95% CI: 32.6–37.5) received at least one prescribed antibiotic. The proportion of prescriptions containing antibiotics was not statistically influenced by patients’ age, profession or level of education. However, it was significantly higher among hospitalized patients (83.4%; 95% CI: 77.1–87.6) than among outpatients (25.1%; 95% CI: 22.7–27.6; *P* < 0.001). Medical specialists and general practitioners were the leading prescribers of antibiotics, followed by medical interns (45.4%, 31.9%, and 30.4%, respectively; *P* < 0.001). Furthermore, antibiotic prescription frequencies varied by the level of healthcare facility, with higher rates observed in tertiary hospitals (36.6%) compared to secondary hospitals (30.7%; *P* = 0.033) (Table 1).

The multivariate analyses showed that compared to medical interns, both specialists (aOR = 0.47; 95% CI: 0.21–0.96; *P* = 0.049) and general practitioners (aOR = 0.19; 95% CI: 0.05–0.73; *P* = 0.019) were significantly less likely to prescribe antibiotics. Regarding the medical wards, patients treated in the emergency ward had a substantially higher risk of receiving at least one antibiotic (aOR = 8.35; 95% CI: 4.77–15.0; *P* < 0.001) compared to patients treated in general medicine. Those managed in the infectious diseases department were significantly less likely to receive antibiotics (aOR = 0.44, 95% CI: 0.22–0.87; *P* = 0.017). Finally, hospitalized patients had more than 23 times the odds of being prescribed an antibiotic compared to outpatients (aOR = 23.8; 95% CI: 15.9–36.7; *P* < 0.001) (Table 2).

**Table 2.** Multivariable logistic regression analysis of factors associated with antibiotic prescribing in Conakry, Guinea (June-October 2024)

Characteristic	Unadjusted OR (95 % CI)	*P*-value	Adjusted OR (95 % CI)	*P*-value
Patient Sex				
Female	—	—	—	—
Male	1.27 (1.02 − 1.57)	0.030	1.21 (0.91 − 1.61)	0.2
Patient Marital status				
Single	—	—	—	—
Married	0.80 (0.64 − 1.00)	0.047	0.91 (0.67 − 1.24)	0.6
Patient status				
Outpatient	—	—	—	—
Inpatient	15.0 (10.6 − 21.7)	< 0.001	23.7 (15.8 – 36.5)	< 0.001
Prescriber				
Medical interns	—	—	—	—
Specialist physician	1.90 (1.35 – 2.71)	< 0.001	0.47 (0.21 – 0.96)	0.049
General practitioner	1.07 (0.78 – 1.47)	0.700	0.19 (0.05 – 0.73)	0.019
Prescribers’ experience				
≤5 years	—	—	—	—
>5 years	1.18 (0.93 – 1.50)	0.2	0.94 (0.59 – 1.48)	0.8
Hospital ward				
General medicine	—	—	—	—
Infectious diseases	1.27 (0.74 – 2.24)	0.4	0.44 (0.22 – 0.87)	0.017
Emergency	2.90 (1.78 – 4.87)	< 0.001	3.68 (2.08 – 6.70)	< 0.001
Dermatology	1.58 (0.96 – 2.69)	0.082	0.33 (0.07 – 1.41)	0.15
Gynaecology	2.32 (1.41 – 3.92)	0.001	1.72 (0.97 – 3.11)	0.067
Traumatology	4.22 (2.60 – 7.10)	< 0.001	0.67 (0.17 – 2.41)	0.6

Data expressed by wards shows that empirical prescribing was the leading practice in all wards, representing a total proportion of 99.4% (516/519). Except for the gynaecology, medicine and infectious diseases wards, prescribing a single antibiotic was significantly predominant. The grouped proportion of prescriptions containing a single antibiotic line was 75.5%. The route of administration varied from ward to ward, with an overall predominance of the oral route at 62.2% (323/519), followed by the parenteral route at 41.4% (215/519). In all wards and according to the WHO AWaRe classification, the majority of prescribed antibiotics belonged to the Access group, at 68.2% (354/519), followed by the Watch group, at 42.1% (219/519). No antibiotics were prescribed to the Reserve group.

The most common clinical indications or reasons for antibiotic prescription were fractures of the knee, ulna or bones (19.3%), skin infections and diseases (13.9%), gastrointestinal infections (9.8%), gynaecological and obstetric infections (9.5%) and malaria (5.8%). However, a considerable proportion (31.2%) of antibiotic prescriptions were associated with other, unspecified reasons (Table 3).

**Table 3.** Prevalence and characteristics of prescribed antibiotics by hospital wards in Conakry, Guinea (June–October 2024)

		Antibiotics Prescription Department						
Characteristics	*n* = 519	Dermatology *n* (%)	Emergency *n* (%)	Gynaecology *n* (%)	Infectious diseases *n* (%)	Medicine *n* (%)	Traumatology *n* (%)	*P* value
Number of antibiotics per patient								<0.001
One antibiotic	388 (75.5)	71 (18.3)	93 (23.97)	37 (9.5)	18 (4.6)	21 (5.4)	148 (38.1)	
Two antibiotics	109 (21.2)	9 (8.3)	21 (19.3)	57 (52.3)	20 (18.3)	2 (1.8)	0 (0.0)	
Three antibiotics and more	17 (3.3)	0 (0.0)	3 (17.6)	4 (23.5)	9 (52.9)	1 (5.9)	0 (0.0)	
Type of treatment								0.11
Directed	3 (0.6)	0 (0.0)	0 (0.0)	0 (0.0)	0 (0.0)	1 (33.3)	2 (66.7)	
Empirical	516 (99.4)	80 (15.5)	121 (23.4)	99 (19.2)	47 (9.1)	23 (4.5)	146 (28.3)	
WHO AWaRe classification								
Access group	354 (68.2)	50 (14.2)	80 (22.7)	82 (23.2)	36 (10.2)	16 (4.5)	89 (25.2)	0.004
Watch group	219 (42.2)	38 (16.6)	65 (28.4)	26 (11.3)	26 (11.3)	12 (5.2)	62 (27.0)	<0.001
Patient status								<0.001
Outpatient	308 (59.3)	79 (25.6)	120 (39.0)	54 (17.5)	9 (2.9)	22 (7.1)	24 (7.8)	
Inpatient	211 (40.7)	1 (0.5)	1 (0.5)	45 (21.3)	38 (18.0)	2 (0.9)	124 (58.8)	
Route of administration								
Oral	323 (62.2)	76 (23.5)	66 (20.4)	94 (29.1)	21 (6.5)	19 (5.8)	47 (14.5)	<0.001
Parenteral	215 (41.4)	1 (0.5)	70 (32.6)	1 (0.5)	33 (15.3)	7 (3.2)	103 (47.9)	<0.001
Others	29 (5.6)	11 (37.9)	2 (6.9)	14 (48.3)	2 (6.9)	0 (0.0)	0 (0.0)	<0.001
Reason for prescription								<0.001
Fractures of the knee, ulna or bones	100 (19.3)	0 (0.0)	7 (7.0)	0 (0.0)	0 (0.0)	0 (0.0)	93 (93.0)	
Gastrointestinal infections	51 (9.8)	0 (0.0)	42 (82.3)	0 (0.0)	2 (3.9)	7 (13.7)	0 (0.0)	
Pulmonary Infections	7 (1.3)	0 (0.0)	3 (42.8)	0 (0.0)	0 (0.0)	4 (57.1)	0 (0.0)	
Infections of the central nervous system	5 (0.9)	0 (0.0)	0 (0.0)	0 (0.0)	5 (100.0)	0 (0.0)	0 (0.0)	
Malaria	30 (5.8)	0 (0.0)	20 (66.7)	0 (0.0)	9 (30.0)	1 (3.3)	0 (0.0)	
Obstetric or gynaecological infections	49 (9.5)	0 (0.0)	0 (0.0)	44 (89.8)	4 (8.2)	1 (2.0)	0 (0.0)	
Other reasons are not clearly defined	162 (31.2)	11 (6.8)	29 (17.9)	51 (31.5)	20 (12.3)	7 (4.3)	44 (27.1)	
Otolaryngology infections	6 (1.2)	0 (0.0)	6 (100.0)	0 (0.0)	0 (0.0)	0 (0.0)	0 (0.0)	
Sexually transmitted diseases	13 (2.5)	0 (0.0)	4 (30.7)	1 (7.7)	5 (38.4)	3 (23.0)	0 (0.0)	
Skin diseases, infectious	72 (13.9)	69 (95.8)	2 (2.7)	1 (1.4)	0 (0.0)	0 (0.0)	0 (0.0)	
Surgical site infections	12 (2.3)	0 (0.0)	1 (8.3)	0 (0.0)	0 (0.0)	0 (0.0)	11 (91.7)	
Influenza	3 (0.5)	0 (0.0)	2 (66.6)	1 (33.3)	0 (0.0)	0 (0.0)	0 (0.0)	
Intra-abdominal sepsis	3 (0.5)	0 (0.0)	1 (33.3)	0 (0.0)	2 (66.7)	0 (0.0)	0 (0.0)	

### Characteristics of antibiotics prescribed by the WHO AWaRe classification groups

Analysis of all prescriptions containing at least one antibiotic line, according to the WHO AWaRe classification, showed that the proportion of outpatients who received Access group antibiotics (67.5%) was lower compared to inpatients (68.7%), with *P* < 0.010. However, for antibiotics in the Watch group, the prescribing trend was higher for outpatients, 44.5% versus 38.9% for inpatients; *P* = 0.084. Secondary hospitals prescribed Access group antibiotics predominantly (80.5%), whereas the proportion was lower in tertiary hospitals (64.4%). Conversely, Watch group antibiotics were more frequently prescribed in tertiary-level hospitals (45.7%) than in secondary hospitals (30.9%), *P* < 0.001. General practitioners were the highest prescribers of Access antibiotics (74.2%). Whereas medical interns (34.3%) frequently prescribed antibiotics from the Watch group compared to specialists (43.0%) and general practitioners (43.5%), the difference was statistically significant (*P* < 0.001).

Regarding the route of administration, a higher proportion of Access antibiotics (81.4%) were prescribed orally, whereas antibiotics administered parenterally were more often from the Watch group (63.3%; *P* < 0.001). Prescriptions for ordinary and routine infections, such as gastrointestinal infections (70.6%), pulmonary diseases (100%), sexually transmitted infections (76.9%) and other reasons not clearly defined, predominantly involved Access antibiotics, and Watch group antibiotics were prescribed mainly for reasons, such as intra-abdominal sepsis, malaria, and gynaecological diseases (Table 4).

**Table 4.** Characteristics of prescribed antibiotics by the WHO AWaRe classification in Conakry, Guinea (June–October 2024)

Variable	WHO AWaRe classification of antibiotics					
	Access group			Watch group		
	No *n* (%)	Yes *n* (%)	*P* value	No *n* (%)	Yes *n* (%)	*P* value
Patient status			0.010			0.084
Inpatient	66 (31.3)	145 (68.7)		129 (61.1)	82 (38.9)	
Outpatient	99 (32.1)	209 (67.9)		171 (55.5)	137 (44.5)	
Hospital type			<0.001			<0.001
Secondary hospital	24 (19.5)	99 (80.5)		85 (69.1)	38 (30.9)	
Tertiary hospital	141 (35.6)	255 (64.4)		215 (54.3)	181 (45.7)	
Prescriber			0.009			0.4
Medical interns	26 (37.1)	44 (62.9)		46 (65.7)	24 (34.3)	
General practitioner	73 (25.8)	210 (74.2)		160 (56.5)	123 (43.5)	
Specialist	65 (39.4)	100 (60.6)		94 (56.9)	71 (43.0)	
Route of administration						
Oral	60 (18.6)	263 (81.4)	<0.001	220 (68.1)	103 (31.9)	<0.001
Parenteral	103 (47.9)	112 (52.1)	<0.001	79 (36.7)	136 (63.3)	<0.001
Others	8 (27.6)	21 (72.4)	0.8	19 (65.)	10 (34.4)	0.5
Reason for prescription			<0.001			<0.001
Fractures of the knee, ulna or bones	45 (45.0)	55 (55.0)		53 (53.0)	47 (47.0)	
Gastrointestinal infections	15 (29.4)	36 (70.6)		22 (43.1)	29 (56.9)	
Pulmonary infections	0 (0.0)	7 (100.0)		7 (100.0)	0 (0.0)	
Infections of the central nervous system	2 (40.0)	3 (60.0)		1 (20.0)	4 (80.0)	
Malaria	10 (33.3)	20 (66.7)		12 (40.0)	18 (60.0)	
Obstetric or gynaecological infections	17 (34.7)	32 (65.3)		19 (38.8)	30 (61.2)	
Other reasons are not clearly defined	31 (19.1)	130 (80.9)		116 (70.6)	46 (28.4)	
Otolaryngology infections	4 (66.7)	2 (33.3)		4 (66.7)	2 (33.3)	
Sexually transmitted diseases	3 (23.1)	10 (76.9)		8 (61.5)	5 (38.5)	
Skin diseases, infectious	28 (38.9)	44 (61.1)		46 (63.9)	26 (36.1)	
Surgical site infections	5 (41.7)	7 (58.3)		7 (58.3)	5 (41.7)	
Influenza	1 (33.3)	2 (66.7)		1 (33.3)	2 (66.7)	
Intra-abdominal sepsis	1 (33.3)	2 (66.7)		0 (0.0)	3 (100.0)	

### Distribution of antibiotic classes prescribed

**Figure 1.**
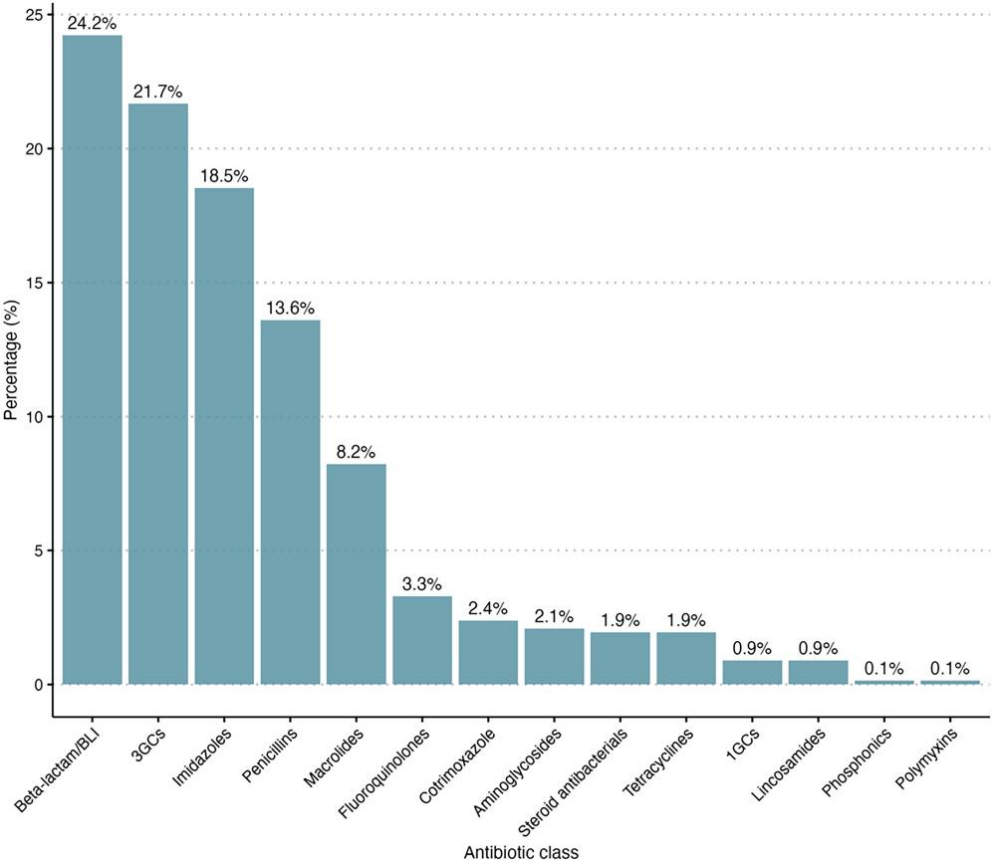
Proportion of prescribed antibiotics class in six Conakry hospital wards in Conakry, Guinea (June–October 2024).

The most frequently prescribed antibiotic classes in the hospital facilities included were beta-lactams/beta-lactamase-inhibitor (24.2%, 162/669), followed by third generation cephalosporins (21.7%, 145/669), imidazoles, particularly metronidazole (18.5%, 124/669) and penicillins (13.6%, 91/669) [Figure 1, Table S1 (available as Supplementary data at *JAC-AMR* Online)].

**Figure 2.**
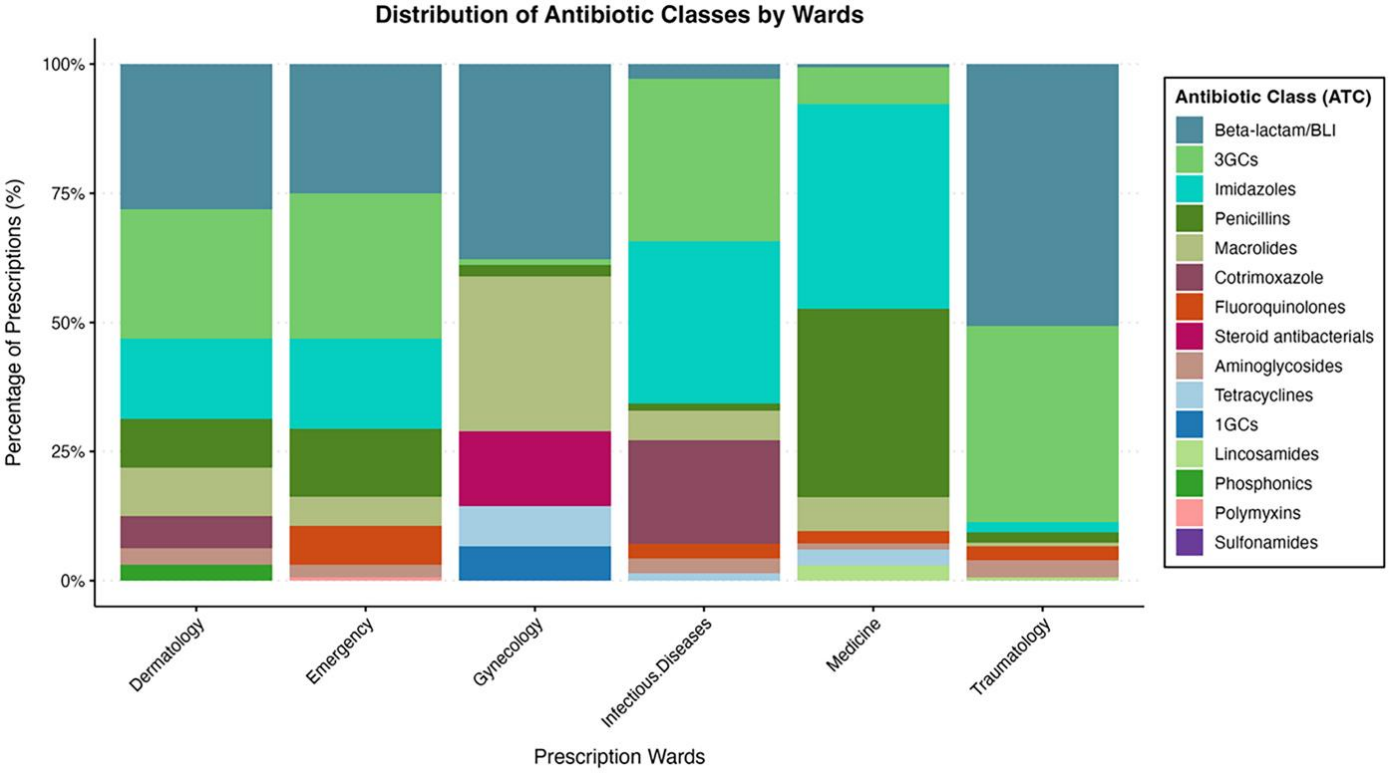
Proportion of prescribed antibiotic classes in Conakry hospitals by prescription ward in Conakry, Guinea, from June to October 2024 (*P* value < 0.0001; Cramer’s *V* = 0.461).

**Figure 3.**
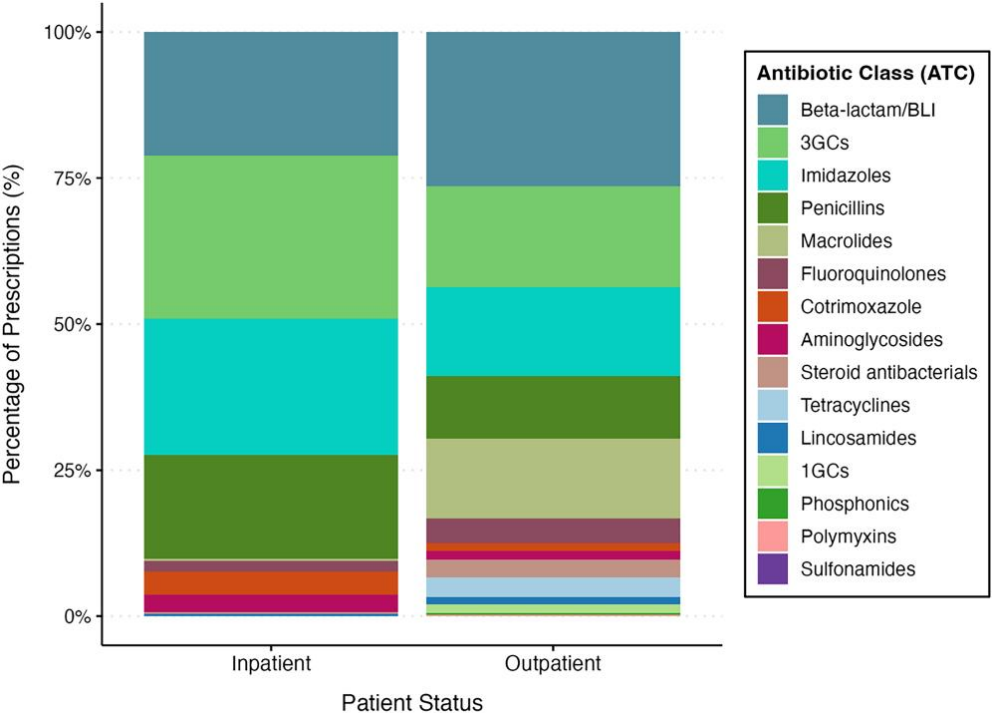
Proportion of prescribed antibiotic classes in Conakry hospitals by participants’ status, from June to October 2024 (*P* value < 0.0001; Cramer’s *V* = 0.3648).

The distribution of prescribed antibiotic classes varied considerably between hospital wards. beta-lactams/beta-lactamase inhibitors, 3GCs, were the most commonly prescribed antibiotics in the majority of the hospital wards, followed by imidazoles. On the other hand, penicillins are the second most widely used antibiotics in general medicine wards, while macrolides are commonly used in gynaecology (Figure 2). Among inpatients, prescriptions were dominated by beta-lactams/beta-lactamase–inhibitor combinations, imidazoles, and 3GCs. In contrast, outpatient prescriptions exhibited greater diversity, with higher proportions of macrolides, fluoroquinolones and cotrimoxazole (Figure 3). A Fisher's exact test with a Monte Carlo simulation revealed a significant association between patient status (inpatient versus outpatient) and hospital wards versus the distribution of the prescribed antibiotics (*P* < 0.0001). The magnitude of these associations, as measured by Cramer’s *V*, was moderate for patient status (*V* = 0.36) and strong for hospital wards (*V* = 0.46), indicating that antibiotic class prescription varies substantially according to both hospitalization status and clinical service.

## Discussion

Evidence on the prevalence of antibiotic prescribing and the most commonly used classes of antibiotics in hospitals is essential for each country and health system. This enables the assessment of the level of antibiotic consumption and measures the impact of initiatives aimed at promoting appropriate antibiotic use. In this study, we conducted a point prevalence survey to evaluate antibiotic use and compliance with WHO guidelines in public hospitals in Conakry, the capital of the Republic of Guinea.

Among the 1482 patients included, 35% were receiving at least one antibiotic; this proportion reached 83% among hospitalized patients. The global rate observed in our study is above that reported in South Korea (14.1% of all patients, with 50.8% of prescriptions relating to inpatients) and 21.1% in Malaysia.^26,27^

Among outpatients, the antibiotic prescription rate is 25.1%. These figures are favourable compared to the international norm, which suggests that less than 30% of consultations should result in the prescription of antibiotics.^28,29^ Also, this proportion observed in our study is lower than those reported in other similar studies.^30–32^ This comparatively low rate could be explained by the specific medical wards included and the level of consultation in our study.

For inpatients, WHO recommends that the prescription of antibiotics should be 40% or less.^24^ However, our data show that this proportion is doubled for patients treated on an inpatient basis in Guinea. Similar trends can be observed in other countries, including Benin, which has 82.9%, Ghana, with 68.5%, Zambia (75%), Ethiopia (65%) and other West African countries.^33–37^ Some countries have reported lower rates of antibiotic prescribing among hospitalized patients, suggesting that significant efforts have been made to ensure more rational prescribing. This is the case in South Africa (33.6%), Tanzania (47%) and Senegal (45.5%).^6,38,39^

The high prevalence of antibiotic prescribing among inpatients could be explained by factors such as the lack of targeted education for physicians and difficulties in accessing laboratories and microbiological results. Also, hospitalized patients are often in a more serious clinical condition, and when faced with a patient with an infectious syndrome, doctors prescribe antibiotics even without laboratory results. Furthermore, other factors that may be personal, psychological and organizational have been reported,^40^ including work experience, knowledge, availability of and compliance with guidelines, perceived pressure from patients and explicit requests for antibiotics by patients.^41,42^

The most commonly prescribed antibiotics are beta-lactams/beta-lactamase inhibitors, 3GCs, imidazoles (metronidazole), penicillins and macrolides. Other studies also highlighted the everyday use of these antibiotics.^14,27,43^ Moreover, a systematic review of 17 studies of patterns of antimicrobial prescription in Africa also reported beta-lactam penicillin (amoxicillin, clavulanic acid) and 3GCs as the most commonly used antibiotics.^44^ The 23-fold greater odds of receiving at least one antibiotic for hospitalized patients could essentially be attributable to the nature of illness, diagnostic processes, comorbidities and prophylaxis.

In addition, almost all antibiotic prescriptions were empirical, with no prior biological investigations. Bacterial culture and antibiotic susceptibility testing were requested for only 3 patients. This finding has also been reported in several other studies.^8,15,33,45^ Knowing that this practice could significantly increase the risk of AMR development, particularly in the context of the frequent prescription of broad-spectrum antibiotics such as ceftriaxone, cefixime, azithromycin and clarithromycin. It should be noted that studies in Guinea have reported a high prevalence of bacteria resistant to broad-spectrum antibiotics, such as *Escherichia coli*, *Klebsiella pneumoniae*, *Staphylococcus aureus* and *Pseudomonas aeruginosa*, resistant to 3GCs and carbapenems.^46,47^ These prescription practices, especially in low- and middle-income countries, can often be driven by the clinical urgency or limited access to adequately equipped diagnostic laboratories. Moreover, the noted reasons for antibiotic prescribing, such as malaria, influenza, or other reasons that are not clearly defined, highlight the urgent need for awareness-raising actions and a solid surveillance system to be implemented in the Guinea hospital system.

According to the WHO AWaRE classification, 64.3% of the antibiotics prescribed fall under the Access group, and 33.3% fall under the Watch group. In the rural healthcare district of Forécariah in Guinea, a previous study reported 73.6% for Access and 27.6% for Watch antibiotic prescription for febrile patients.^14^ In Tanzania, authors reported proportions of 60.8% and 33.3%, respectively, for the Access and Watch groups.^8^ Although the current proportion of Access group antibiotics (64.3% of prescriptions) aligns with the WHO minimum target of ≥60% for antibiotic consumption,^48^ it is vital to continue efforts to achieve the ambitious new goal (≥70%) of the Global multisectoral action needed to reach targets by 2030.^49^ Concurrently, the proportion of Watch group antibiotics (33.3% of prescriptions) represents an area requiring significant action. Limiting their use is imperative, as these antibiotics are under close surveillance due to their high potential to promote the emergence and spread of antimicrobial resistance.

We also found that antibiotic prescribing differed significantly by hospital type, with secondary hospitals predominantly prescribing antibiotics for the Access group, whereas the proportion of Watch antibiotics was higher in tertiary hospitals.

These findings further underscore that antibiotic prescribing practices may vary considerably across healthcare settings and are influenced by hospital type and prescribers’ experience. Consequently, interventions aimed at reducing antibiotic overuse and antimicrobial resistance should be carefully tailored and context-specific.

In Guinea, a Multisectoral National Action Plan against antimicrobial resistance was developed in 2020 within a One Health framework and aims to improve coordination across the human, animal and environmental health sectors, strengthen antimicrobial use surveillance, and build laboratory capacity for AMR detection.^12^ However, unlike in some other health systems, there is no publicly available evidence of established and functional AMS committees at either the national or hospital level, nor of national antibiotic prescribing guidelines. These contextual gaps, combined with challenges such as limited infection prevention and control measures, limited access to microbiology laboratory and resource constraints, likely contribute to the patterns of empirical prescribing observed in our study. In this way, AMS programs should be implemented, particularly in tertiary hospitals where antibiotic use is more common.

This study has some limitations. Firstly, it did not cover all hospital wards; key departments, such as paediatrics or surgery, were not included, which limits the generalizability of the findings to the entire hospital population. Secondly, its cross-sectional design does not allow an assessment of whether the antibiotics prescribed were used by the patients and does not provide information on the clinical appropriateness of the prescriptions.

Despite these limitations, the study also has notable strengths. It included a large sample of more than 1400 patients, which increased the statistical power and reliability of the analysis. It was also based on a standardized WHO methodology, which ensured good data collection and allowed the indicators obtained to be compared with those reported in the regional and international literature.

### Conclusion

This study highlights a high prevalence of antibiotic prescriptions in hospital settings in Conakry, Guinea, which is particularly pronounced among hospitalized patients compared with outpatients. Our findings suggest an urgent need to improve antibiotic prescription practices, strengthen diagnostic capacities, educate physicians and patients, and implement antimicrobial stewardship programs in hospitals. These strategies align with national policies and regional stewardship frameworks, are crucial for reducing empirical antibiotic prescribing practices, preserving antibiotic effectiveness, and preventing the emergence of new antimicrobial resistance and the public health threats it poses.

## Supplementary Material

dlaf223_Supplementary_Data

## Supplementary data

Table S1 is available as Supplementary data at *JAC-AMR* Online.
