# Supplementary material for: Prevalence and patterns of antibiotic prescriptions in Conakry hospitals, Guinea: a multicentre cross-sectional survey
Source: JAC Antimicrob Resist. 2025 Nov 20;7(6):dlaf223. doi: 10.1093/jacamr/dlaf223 (PMC12631128; doi:10.1093/jacamr/dlaf223)
Supplement: dlaf223_Supplementary_Data [file dlaf223_supplementary_data.docx]

**Supplementary data**

**Table S1: Frequency of antibiotics prescribed for participants at six Conakry hospital wards (n=669)**

| **Antibiotic agent** | **Prescribing**  **frequency** | **%** | **AWaRe classification** | **ATC Classification** |
| --- | --- | --- | --- | --- |
| Amoxicillin and clavulanic acid | 162 | 24.2 | Access | Beta-lactam combination penicillin |
| Ceftriaxone | 126 | 18.8 | Watch | 3rd generation cephalosporins |
| Metronidazole | 111 | 16.6 | Access | Imidazoles |
| Amoxicillin | 84 | 12.6 | Access | Extended-spectrum penicillins |
| Azithromycin | 34 | 5.1 | Watch | Macrolides |
| Cefixime | 18 | 2.7 | Watch | 3rd generation cephalosporins |
| Cotrimoxazole | 16 | 2.4 | Access | Sulphamethoxazole-Trimethroprim |
| Ciprofloxacin | 14 | 2.1 | Watch | Fluoroquinolones |
| Doxycycline | 13 | 1.9 | Access | Tetracyclines |
| Fusidic acid (non-systemic) | 13 | 1.9 | N/A* | Steroid antibacterials |
| Clarithromycin | 10 | 1.5 | Watch | Macrolides |
| Gentamicin | 9 | 1.3 | Access | Aminoglycosides |
| Tinidazole | 7 | 1.0 | Access | Imidazoles |
| Clindamycin | 6 | 1.2 | Access | Lincosamides |
| Secnidazole | 6 | 1.2 | Access | Imidazoles |
| Spiramycin | 6 | 1.2 | Watch | Macrolides |
| Cefadroxil | 6 | 0.9 | Access | 1st generation cephalosporins |
| Levofloxacin | 5 | 0.7 | Watch | Fluoroquinolones |
| Ampicillin | 4 | 0.6 | Access | Extended-spectrum penicillins |
| Erythromycin | 4 | 0.6 | Watch | Macrolides |
| Amikacin | 3 | 0.4 | Access | Aminoglycosides |
| Troleandomycin | 1 | 0.1 | Watch | Macrolides |
| Ofloxacin | 2 | 0.3 | Watch | Fluoroquinolones |
| Neomycin (non-systemic) | 2 | 0.3 | N/A* | Aminoglycosides |
| Phenoxymethylpenicillin | 1 | 0.1 | Access | Beta-lactamase-sensitive penicillins |
| Ceftizoxime | 1 | 0.1 | Watch | 3rd generation cephalosporins |
| Norfloxacin | 1 | 0.1 | Watch | Fluoroquinolones |
| Fosfomycin | 1 | 0.1 | Watch | Other antibacterials |
| Polymyxin b (non-systemic) | 1 | 0.1 | N/A* | Polymyxins |
| Cloxacillin | 1 | 0.1 | Access | Penicillins |
| Flucloxacillin | 1 | 0.1 | Access | Beta-lactamase-resistant penicillins |

ATC: Anatomical Therapeutic Chemical,

N/A: Not applicable for AWaRe classification
